# Designing a systemic intervention for student loneliness and social connectedness using a mixed-methods, co-creation approach

**DOI:** 10.1038/s44184-026-00191-9

**Published:** 2026-02-21

**Authors:** Sophie R. Homer, Madison Milne-Ives, Emily Cornford, Rebecca Richardson, Alvise Rogers, Onshell Relf, Jackie Andrade, Edward Meinert, Jon May

**Affiliations:** 1 School of Psychology, Faculty of Health, University of Plymouth, Plymouth, UK; 2 Translational and Clinical Research Institute, Newcastle University, Newcastle upon Tyne, UK; 3 Centre for Health Technology, School of Nursing and Midwifery, University of Plymouth, Plymouth, UK; 4 Strategy, Planning and Analytics, University of Plymouth, Plymouth, UK; 5 Department of Primary Care and Public Health, School of Public Health, Imperial College London, London, UK

**Keywords:** Psychology, Human behaviour

## Abstract

Loneliness and social (dis)connectedness are significant public health concerns, particularly among university students. Despite calls to reconceptualise loneliness as a systemic issue, interventions typically target individual students. This series of studies used a sequential mixed-methods and participatory action approach to explore students’ social experiences and co-design a digital health solution. Focus groups (Study One) and a survey (Study Two) revealed that students see universities as partly responsible for their social connectedness, with perceptions of campus space being key. These insights informed the co-design of MAPP (Study Three), a preventative, system-focused digital solution. MAPP is an interactive campus map that visualises the university’s living social network. It increases the visibility and accessibility of the university community to foster belonging, scaffold social engagement, and support institutional inclusivity. By shifting focus from the lonely student to the university as a social system, MAPP offers a novel, holistic response to student loneliness.

## Introduction

Social connectedness is not a privilege—it is a necessity. We all need to feel like we belong^1^, and meaningful relationships are essential for happiness, general wellbeing, and, for university students, educational success^2,3^. The UK university experience is often associated with community, connection, and easy access to social networks^4^. Yet, more than one-third of students experience loneliness^5–8^. Existing solutions typically seek to identify and treat lonely students^9^, but we argue that loneliness is not an individual problem—it is a social one. This project investigated students’ experiences of loneliness and social connectedness and co-designed a digital health solution that takes a systemic, rather than individualistic, approach.

Loneliness and social isolation are major public health concerns^10^, that have reached ‘epidemic’ levels according to NIHR^11^. They are associated with both mental and physical ill-health^5,12,13^, likely in a bi-directional relationship^14^. For example, loneliness and depression reciprocally reinforce one another^15^. In other words, the worse one feels, the lonelier one becomes, and the lonelier one becomes, the worse one feels.

Though related, loneliness and social isolation are distinct concepts. Social isolation describes a lack of social ties, while loneliness is the negative, subjective *perception* that one’s social connections are lacking in quantity and/or quality^16,17^. Therefore, loneliness is uniquely characterised by the negative emotional experience of disconnection. This conceptualisation aligns with the evolutionary theory of loneliness, which frames loneliness as an adaptive signal to reconnect socially^10^. Although both loneliness and social isolation independently predict mental and physical ill-health^13,18^, there are gender differences. Social isolation, but not loneliness, predicts mental ill-health in males while both predict mental ill-health in females^19^. Therefore, we must consider both concepts in tandem^16^, as well as their impacts on different social groups^14^.

University students increasingly experience loneliness and social isolation. Between 25–75% of students experienced loneliness before the COVID-19 pandemic^5,6,8^, which exacerbated these issues^20^, particularly among younger people^20–22^. Transitioning to new environments and away from existing social networks increases students’ risk of experiencing loneliness and social isolation^5,23^, which in turn can lead to increased risk behaviours, drug use, self-harm, suicide, and future unemployment^2^. In response, a priority of the University Mental Health Charter is to improve social integration and belonging^24^, and Universities UK’s seminal Stepchange Framework calls for a whole-university response^25^.

Student loneliness has been addressed in multiple ways. A recent review^9^ identified 37 interventions, which included psychoeducation (*n* = 12), mindfulness (9), social opportunities (8), and support groups (5). Digital health interventions have great potential for success, particularly in a population so used to technology^26,27^, for whom support services are increasingly strained^28^. Yet, only five interventions were delivered digitally: a depression support forum^29^; facilitated online chat^30,31^; an online journal^30^; and one app, Nod^26^, which delivers mindfulness activities and sociability tips. Outside of the review, Nod’s competitors include Noneliness—a gamified, social-media-like interface allowing users to chat, view events, and join groups^32^—and Connect + , a 6-week positive psychology programme^33^.

There are several drawbacks to existing loneliness interventions. Firstly, they are reactive, in that they target existing problems. Rather than intervening once the problems are established, it would be preferable to prevent loneliness and social isolation in the first place. Secondly, their approach is individualistic, treating loneliness as an individual problem to be solved by targeting lonely individuals. This could be problematic because (i) students do not want to identify as lonely individuals^7^ due to stigma^14^, and (ii) individualistic interventions do not sufficiently address the social and systemic dimensions of loneliness and social isolation.

These social and systemic dimensions are receiving increasing research attention. Scholars argue that loneliness is an issue of human rights^34^ and social justice^14^, caused by systemic oppression^35,36^. At least three psychological and sociological theories support a systemic view of loneliness and social isolation. Self-determination theory^1^ identifies belonging to a larger community as essential for psychological health. Symbolic interactionism theory^37^ emphasises the importance of shared meanings and symbols within those communities. Social capital theory^38^ highlights the benefits of access to shared networks and resources. Without communities, complete with shared meanings, symbols, and accessible resources, individuals can feel disconnected and lonely. Moreover, marginalised groups (i.e., those most disadvantaged by power systems) experience higher levels of loneliness^14,39^. Despite this compelling theoretical and empirical rationale, responses to student loneliness tend to overlook systemic considerations. Instead, they deliver interventions to lonely individuals or groups without addressing the wider systems and communities within which they exist.

Therefore, there is a need for a digital solution that overcomes these drawbacks by taking a whole-population, preventative, and systemic approach to student loneliness. The solution cannot be social media, which is associated with depression, low-self-esteem, body-image concerns^40–42^ and loneliness, especially when used purposely for social connection^43^. This project drew upon the principles of Participatory Action Research—an inclusive approach that actively involves the stakeholder community as co-researchers and emphasises collaboration and co-creation—to:

i. Enable students to explore experiences of loneliness and social connectedness at university;
ii. Co-design a digital solution, taking a preventative, whole-population, systemic approach.

## Methods (Studies One–Three)

### Participatory action research

We adopted principles of participatory action research^44,45^ and user-centred design^46^ to maximise co-production with end users. Three paid Undergraduate Research Assistants (URAs) and one Postgraduate Research Fellow co-led the project.

### User-centred design

**Fig. 1:**
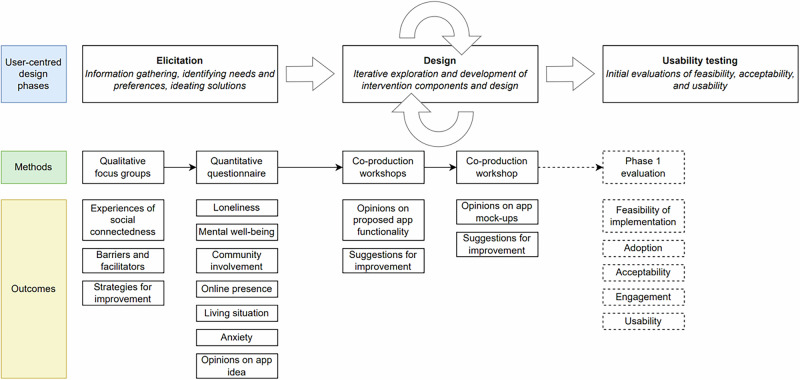
User-centred design process for developing a systemic intervention to support student social connectedness. This figure illustrates the user-centred design process used to develop a systemic intervention to support student social connectedness and address loneliness. The process is structured into three key phases: elicitation, design, and usability testing. The elicitation phase involved gathering information on student needs, preferences, and experiences through qualitative focus groups and a quantitative questionnaire. The design phase involved co-production workshops where students provided feedback on proposed app functionality and mock-ups, with iterative refinement based on participant input. The usability testing phase (planned as the next stage) will focus on evaluating feasibility, adoption, acceptability, engagement, and usability of the intervention. Arrows indicate the sequential flow and iterative feedback loops within the design phase. No abbreviations, colours, or symbols requiring further definition are used in this figure.

User-centred design^46^ involves three phases: elicitation, design, and usability testing. This project covered phases one and two (Fig. 1).

In the elicitation phase, we used a mixed-methods approach to explore students’ experiences of loneliness and social connectedness at university and identify needs. We conducted focus groups (Study One; *n* = 13) on experiences of social (dis)connectedness, followed by a larger-scale survey (Study Two, *n* = 42) to interrogate the generalisability of focus group themes and gather feedback on our initial conceptualisation of MAPP. In the design phase, findings from the elicitation phase were presented to participants in a series of iterative co-production workshops, where they were used to shape the app’s functional specification and refine an interface design mock-up (Study Three; *n* = 14). Across all studies, 69 participants were recruited (because the survey was anonymous, and not all participants chose to provide demographic information, we are unable to determine whether any participants took part in more than one study). Ethical approval for all studies was obtained from the University of Plymouth, Faculty of Health Research Ethics & Integrity Committee (Project ID 3806). (Note: MAPP is the name we gave to the app during the development process; it is not an acronym but was chosen by the research team to reflect the app’s focus on mapping social opportunities and community connections across the university.)

### Study one methods—focus groups on experiences of social connectedness at university

We conducted focus groups with students to explore, in depth, their perceptions and experiences of loneliness and social connectedness at university.

*Participants:* Students at a UK university were invited via email (student newsletters) and social media to complete an online sign-up form for a study about ‘experiences of university’, in exchange for a £10 shopping voucher. A total of 52 students or recent graduates (<1 year post-graduation) provided demographic data, from which the URAs invited a representative sample of thirteen students to three focus groups (3–5 per group; 6 males, 7 females, mean age = 25.92, SD = 8.85, range: 19–46; see Table 1).

**Table 1:** Study One focus group participant demographics (*n* = 13)

Characteristics	n (%)
Gender	
Male	6 (46.2%)
Female	7 (53.8%)
Age	
Mature student	4 (30.77%)
Not a mature student	9 (69.23%)
Stage of study	
Undergraduate (foundation year)	1 (7.7%)
Undergraduate (second year)	7 (53.8%)
Postgraduate	5 (38.5%)
Mode of study	
Full-time	12 (92.3%)
Part-time	1 (7.7%)
Living situation	
Live alone	3 (23.1%)
Live with family	2 (15.4%)
Live with partner	2 (15.4%)
Live with other students	4 (30.8%)
Live with non-students	1 (7.7%)
Other	1 (7.7%)
Commute to University	
Yes	2 (15.4%)
No	11 (84.6%)
Ethnicity	
White	10 (76.9%)
Asian	1 (7.7%)
Multiple ethnicities	2 (15.4%)
Has a disability	
Yes	5 (38.5%)
No	7 (53.8%)
Prefer not to say	1 (7.7%)
LGBTQ+	
Yes	5 (38.5%)
No	8 (61.5%)
Self-reported social connectedness and satisfaction with social life	
1 (not socially connected and dissatisfied with social life)	1 (7.7%)
2	5 (38.5%)
3	7 (53.8%)
4	0 (0%)
5 (very socially connected and very satisfied with social life)	0 (0%)

*Materials:* With supervision from the Principal Investigator (SH—Lecturer in Psychology at the time), the Undergraduate Research Assistants (URAs) produced a semi-structured topic guide based on existing literature and the team’s prior research experience. The guide explored students’ definitions of social (dis)connectedness, the factors that promote or hinder it, personal experiences of connection and disconnection, and students’ perceptions of how the university could improve social connectedness. The full topic guide is provided as supplementary material.

*Procedure:* Focus groups were conducted via Zoom by two URAs. Participants were briefed and provided informed consent before the recording began. Each group lasted up to one hour and followed a semi-structured format following the topic guide, before participants were debriefed.

*Qualitative data analysis:* We used reflexive thematic analysis following Braun and Clarke’s^47^ six-step approach. Recordings were transcribed verbatim and then coded manually by two URAs. Participant identifiers were assigned retrospectively based on the transcripts. Both URAs independently familiarised themselves with the data and generated preliminary codes. They met regularly (at least weekly) throughout the analysis to review and refine codes collaboratively, resolve discrepancies through discussion and by trialling alternative interpretations, and develop themes, with supervision from the PI provided at least monthly. The URAs’ involvement provided important lived experience that enriched the interpretation of the data. The analysis was iterative: themes were checked against the data and refined by both URAs and the first and second authors until they provided a coherent representation of the data and met Patton’s^48^ criteria for internal homogeneity and external heterogeneity.

### Study two methods—online survey on experiences of social connectedness and initial app ideas

We conducted an online survey to further interrogate Study One’s findings and to gain feedback on the initial conceptualisation of MAPP derived from the conclusions of Study One (specifically, the central, interactive campus map).

*Participants:* Within the same UK university, advertisements for a study about ‘Experiences of university’ were shared via email and social media, linking students directly to the online study. A total of 42 participants completed the survey for the chance to win a share of £100 in shopping vouchers. Thirty-seven provided demographic information (mean age = 24.2, SD = 8.20, range: 18–55; see Table 3).

*Materials:* *Revised UCLA Loneliness Scale*^49^*:* This 20-item scale has participants respond to items including ‘I feel isolated from others’, on a 4-point Likert scale scored 1-4. Scores range from 20 to 80, with scores of 35–49, 50–64, and 65–80 indicating moderate, moderately high, and high levels of loneliness, respectively.

*Short Warwick-Edinburgh Mental Wellbeing Scale (SWEMWBS*^50^*):* This 7-item scale has respondents rate items including ‘I’ve been feeling relaxed’ on a 5-point Likert scale scored 1-5. Scores range from 7 (poorer wellbeing) to 35 (better wellbeing).

*Procedure:* Upon opening the survey, participants were briefed and provided informed consent. They completed demographic questions followed by the UCLA Loneliness Scale and SWEMWBS in a randomised order. Participants then completed free-text and multiple-choice questions on their experiences of social connection, barriers to connection, and perceptions of social media. Lastly, participants evaluated initial conceptualisations of MAPP (as a digital solution for student social connectedness centred around an interactive map of the university campus).

*Data analysis:* The survey yielded both quantitative and qualitative data. Quantitative data were analysed using JASP. Prior to analysis, incomplete responses were removed on the assumption that these participants had withdrawn consent. Descriptive statistics were calculated for all variables, including frequencies and percentages for categorical variables, and means and standard deviations for continuous variables. A Pearson’s correlation coefficient was used to explore the relationship between loneliness (UCLA) and wellbeing (SWEMWBS) scores. No inferential tests were conducted, as the aim of the survey was to describe student experiences to further support the elicitation phase and inform the subsequent co-design phase. Missing data are reported were relevant. Qualitative data (free-text responses) were shaped into narrative summaries to illustrate and provide context for the quantitative findings, a pragmatic approach commonly used to support survey-based research^51,52^.

### Study three methods—iterative think-aloud co-production workshops

**Fig. 2:**
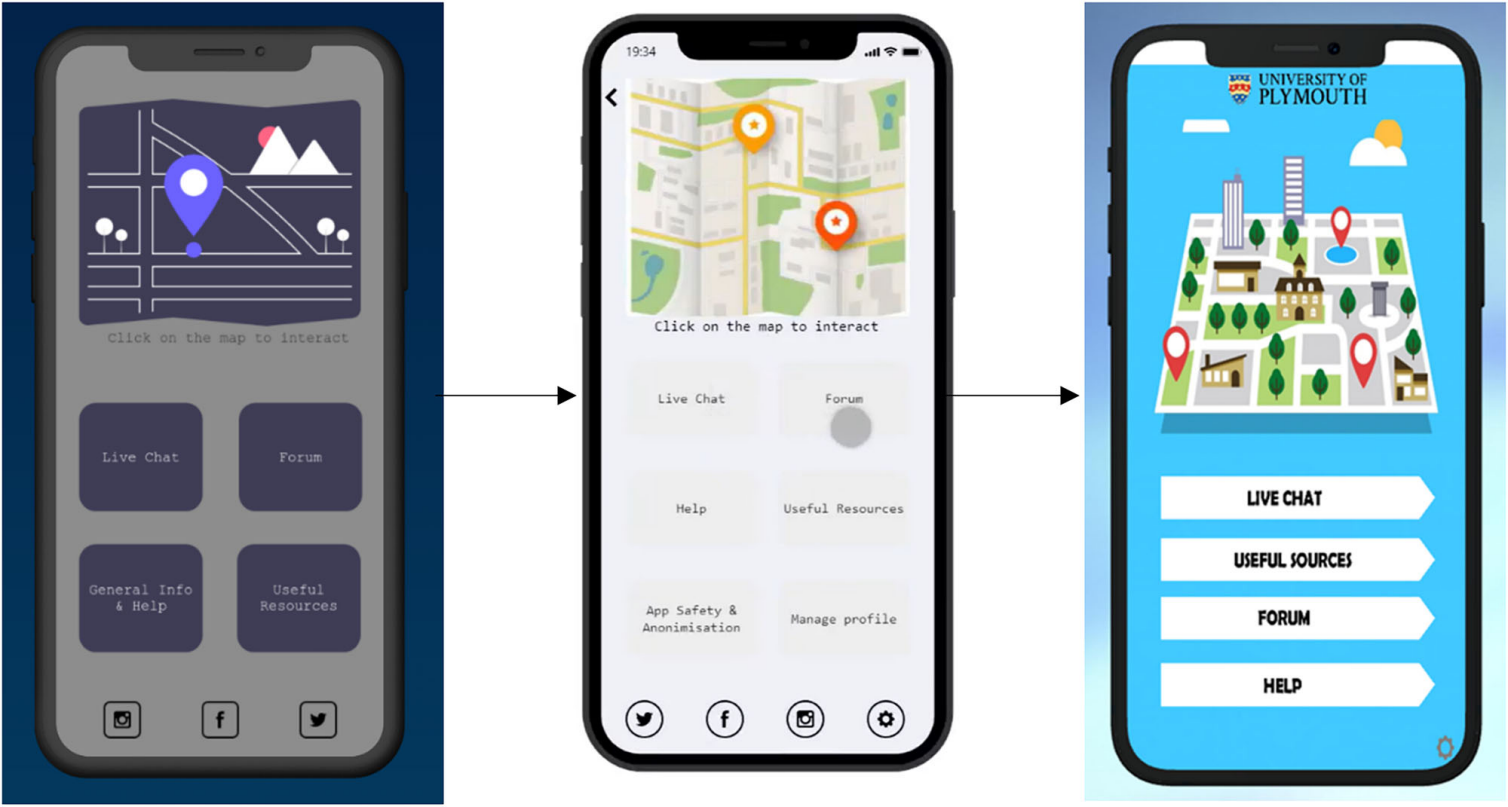
Evolution of the interface design mock-ups produced by the URA (Rogers) based on findings from Study One, Study Two, and the Study Three Co-design workshops. The initial design mock-up (left) and functional mock-up (centre) were created based on functionality and design preferences emerging from Study Two and the Study Three co-design workshops. The design mock-up (right) was subsequently updated in line with students’ preferences for a bright interface following the Study Three mock-up evaluation workshop.

Across two workshops, students co-designed a functional specification for MAPP based on previous results. Using these findings, the URAs produced an interface design mock-up (Fig. 2), which was presented and evaluated in a final workshop and then refined accordingly.

*Participants:* A sample of fourteen students were recruited from the same UK university. Emails (student newsletters) and social media posts (Facebook and Twitter) invited students to complete an online sign-up form for workshops to conceptualise a ‘University social app’. All participants who completed the online sign-up form participated in exchange for a £10 shopping voucher. Nine provided demographic information (mean age = 26, SD = 11.35, range: 21–54; 6 female, 2 male, 1 non-binary; 2 second-year undergraduates, 3 final year undergraduates, 1 placement student, 2 taught postgraduates, 1 postgraduate researcher; 1 part-time student; 8 self-described as white, 1 as mixed/multiple ethnic groups; 5 declared a disability; 3 self-described as LGBTQ + .

*Materials:* Topic guides, produced by the URAs with supervision, facilitated the think-aloud workshops. The guide for workshops 1 and 2 summarised the findings from Studies One and Two and presented initial ideas for the concept, design, and functionality of MAPP. The workshop 3 guide centred around the interface design mock-up.

*Procedure:* Three workshops were conducted with 14 students (3–5 per workshop), each led by one URA. Participants were briefed and provided informed consent before participating. Workshop 1 and 2 participants were asked to consider the findings from Studies One and Two while discussing our initial ideas for the concept, design, and functionality of the app. They built upon these ideas, proposed new ones, and refined them into a functional specification. One URA with previous development experience (Rogers) created an interface design mock-up based on this specification. Workshop 3 participants evaluated and refined the interface design mock-up.

Qualitative data from the co-production workshops were summarised using a descriptive, iterative approach in line with government guidance on think-aloud studies in digital health research^53^. URAs took notes on the co-design workshop proceedings. These notes were reviewed collaboratively by the URAs and wider research team. Key feedback points were identified, grouped thematically, and used by the URAs to produce an initial static app design mock-up (Fig. 2 - left) and interactive functionality mock-up (Fig. 2 - centre). These mock-ups were evaluated by participants in a final mock-up evaluation workshop. URAs took notes from this final evaluation workshop which were summarised separately. The thematic summary process focused on capturing students’ design preferences, usability concerns, and functional requirements. Final themes were agreed through team discussion and checked against the workshop outputs to ensure an accurate representation of student feedback.

## Results (studies one—three)

### Study one results—focus groups on experiences of social connectedness at university

The focus group sample was selected to be as diverse as possible. Table 1 shows the demographic characteristics of the sample.

Focus group data were analysed using thematic analysis (see Methods section for further details). The theme structure is shown in Table 2 and illustrated in the narrative below. Data were organised into three interconnected themes, each with four-six subthemes. Two subthemes spanned all main themes.

**Table 2:** Themes and subthemes

Theme	Subtheme		
1 Experiences of social connection	1.1 Shared experience	Subtheme A: The student’s role	Subtheme B: The university’s role
	1.2 Fitting in and belonging		
	1.3 Connected yet lonely		
	1.4 Fear of missing out and social comparison		
2 Opportunities and ways to connect	2.1 Using social media to avoid anxiety		
	2.2 Connecting with coursemates		
	2.3 The campus as a source of connectedness		
	2.4 Connecting through shared accommodation		
	2.5 Societies and social events		
	2.6 Widening networks		
3 Barriers to connection	3.1 Accessibility and inclusivity		
	3.2 Drinking culture		
	3.3 Problems with accommodation		
	3.4 Interaction anxiety		
	3.5 Mental health as a moderator of connectedness		
	3.6 Shortcomings of social media		

**Theme 1 Experiences of social connection,** *Subtheme 1.1 Shared experience:* Shared experience was central to students’ understanding and experiences of social connectedness. Students placed importance on both the broader university experience—meeting new people from diverse backgrounds, leaving home, trying new things—and specific, non-academic experiences, such as chatting and ‘playing silly games’ (G2, P1) with peers. Hopes and expectations for shared experiences can motivate students to pursue higher education^54^, and are shaped by students’ backgrounds and life experiences:

G2, P1: ‘Quite a large reason of why I went to university was for social interaction was for adult conversation. I’m a Mum…’

*Subtheme 1.2 Fitting in and belonging:* Beyond sharing experiences, ‘fitting in’ seemed necessary in cultivating a sense of belonging. Fitting in involves actively conforming to social norms and behaviours, not just in response to peer pressure, but as a wilful effort to integrate:

G2, P4: ‘I think I would have ended up drinking just to kind of fit in with people.’

This insight demonstrates how students may adopt behaviours that help them feel included, even when these behaviours conflict with their personal preferences or values. Conversely, the pressure to fit in can create feelings of disconnectedness when students are unable or unwilling to engage in normalised behaviours. This tension was particularly evident among mature students, who felt out of sync with the social preferences of younger peers:

G2, P1: ‘The only time I have felt on the limb, if you like, is when it comes to society things or, you know, the kind of party side of it…’

*Subtheme 1.3 Connected yet lonely:* Efforts to fit in can cultivate social connectedness, but connectedness alone does not prevent loneliness:

G1, P2: ‘I think some people think if they’re around people that they won’t be lonely, but it doesn’t mean that does it, they could still feel lonely (…) doesn’t mean they’re connected just because they’re around people.’

Indeed, students described feeling lonely yet being physically surrounded by people and/or participating in shared activities:

G3, P3: ‘I felt very alone, even when I was like in the nightclub, surrounded by people… So I think that I had superficial, you know, sort of social connection. Really, I didn’t feel it.’

This insight suggests that connectedness may operate as a hierarchy, with regular interactions forming the foundation, and deeper emotional connections at higher tiers. Developing superficial interactions into deeper connections can be hindered by challenges such as homesickness, overwhelm, social pressures, or mental ill-health.

*Subtheme 1.4 Fear of missing out and social comparison:* Paradoxically, students’ social connections sometimes heightened their awareness of being excluded from experiences shared by others. One student described ‘the fear that you’re not being invited to certain things’ (G1, P4), while others actually observed friends and acquaintances engaging in activities without them, either in person or through social media:

G3, P4: ‘It just made those feelings of sort of like loneliness worse because I was sort of comparing myself to what could have.’

For students, shared experience is key to social connectedness, and the desire for belonging can lead them to adjust their behaviours to fit in. However, not only is connectedness insufficient to preclude loneliness, it can intensify it through fear of missing out and social comparison. The various ways students establish these connections are explored in *Theme 2*.

**Theme 2 Opportunities and ways to connect,** *Subtheme 2.1 Using social media to avoid anxiety:* Social media plays a dual role in students’ lives by facilitating social connection *(Theme 2)* yet failing to enable deeper connections *(Theme 3)*. For many students, it is the default way to initiate and maintain connections:

G3, P1: ‘Because, like, from my experience, the initial connection is like social media anyway.’

Students may default to social media not only because it is integral to their social lives^27,55^, but also because it enables them to avoid the anxiety of initiating interactions face-to-face:

G3, P1: ‘I feel too anxious to just be like, hey, my name is [NAME]. But on social media… it’s no, like, none of the physical anxiety.’

G3, P5: ‘I think when you make like a friendship online, kind of thing, like, if they don’t reply to you, it’s a little bit less awkward.’

*Subtheme 2.2 Connecting with coursemates:* In terms of in-person interaction, connecting with coursemates was a key way students build connections. Opportunities to connect naturally arise from shared academic activities like groupwork *(Subtheme 1.1)* and from shared lecture spaces:

G1, P3: ‘I think the lecture space was a good area to socialise.’

G2, P1: ‘Have a pleasant time in a lecture with your peers, but then to also go and carry that on campus or off campus for lunch.’

Central to this subtheme is shared space, which extends beyond the classroom in *Subtheme 2.3*.

*Subtheme 2.3 The campus as a source of connectedness:* Beyond academic sessions, students viewed the wider campus as a source of social connectedness. They valued chance encounters with friends, but simply being on campus, even alone, fostered a sense of belonging:

G3, P5: ‘…something that actually is on campus… joins you into the university more and makes you feel more connected with the university. So in a kind of psycho-geography style way.’

The campus’ busy, vibrant atmosphere also contributed to this feeling. Reflecting on the COVID-19 lockdown, some students described the stark contrast when that ambience was missing:

G2, P1: ‘I didn’t expect the change in energy [during lockdown]… this building should be vibrant… full of noise and chatter and friendship and hope…’

Implicit in this subtheme is the expectation that the university should provide social space. This thread is explicit in *Subtheme A: The university’s role*.

*Subtheme 2.4 Connecting through shared accommodation:* Continuing the theme of shared space, accommodation featured as both an opportunity *(Theme 2)* and a barrier *(Theme 3)* to connection. For many, sharing living spaces builds connections organically:

G1, P2: ‘Because we were in halls of residence, everybody was doing things there were things put on.’

G3, P3: ‘I lived in a house of nine of us with my friends so when I came back, like I said, it’s like instantly social.’

However, the quality of these social connections can vary greatly among individuals (see *Subtheme 3.3*).

*Subtheme 2.5 Societies and social events:* In contrast to the more passive experiences of social connectedness described so far, some students actively and intentionally engage with societies and social events:

G3, P2: ‘When the university runs… the little fairs going on, or artwork up in the SU, or coffee mornings. You can just go sit in the morning before lectures and talk to people.’

G2, P3: ‘Go and try and find somewhere you fit in and go find like you know, the big social meetings they have where they have the big tent.’

However, these opportunities are not always perceived as accessible *(Subtheme 3.1)* or appealing, particularly when they involve drinking alcohol (*Subtheme 3.2)*.

*Subtheme 2.6 Widening networks:* Continuing the trend towards passive social involvement, students often socialised within familiar circles, including pre-assigned working groups:

G2, P5: ‘You tend to actually stick with the people you know… you don’t know anyone and you’re kind of hesitant to meet new people…’

Nonetheless, there was a sense that it would be beneficial to cultivate wider networks, which some students achieved through paid or voluntary work:

G2, P4: ‘I volunteer… And that’s been one of my major social things…’

This theme reveals that connectedness can be cultivated passively, by simply belonging to exogenous groups (e.g., course or accommodation), or actively, by joining endogenous groups (e.g., societies) and seeking new social opportunities through wider networks. *Theme 3* explores the obstacles that prevent students from fully engaging with these opportunities.

**Theme 3 Barriers to connection,** *Subtheme 3.1 Accessibility and inclusivity:* Fresher’s Week was seen as the ideal time to make connections – a limited window of opportunity – and some students experienced challenges in accessing social opportunities thereafter. Accessing venues was not always straightforward for disabled students, while commuter students struggled to participate in spontaneous social plans,

G2, P2: ‘…I kind of like, had flashes of like feeling really lonely… I couldn’t have like, these random activities that everybody else was like seeing and doing.’

and others experienced financial barriers,

G2, P3: ‘I can’t afford to do that, like, and then it’s been quite difficult and quite isolated for me…’

These experiences are more than just isolated experiences of inconvenience or disappointment; they have a cumulative effect that can contribute to a general sense of disconnect from the university community.

*Subtheme 3.2 Drinking culture:* Drinking alcohol (excessively) at university has been conceptualised as a social practice—a habitual, socially embedded behaviour^56^ – but it can deter some students from engaging socially,

G2, P4: ‘There’s a lot of pressure to drink. And I think, if you don’t, … it’s very easy to kind of like, become disconnected in that way.’

while others feel forced to conform to drinking norms:

G3, P2: ‘I didn’t have any choice. The only way you could socialise with your society was to get drunk.’

For some, activities involving heavy drinking are unappealing because they involve taking care of intoxicated friends. Consequently, there is a general desire for more alcohol-free social activities in suitable venues:

G2, P1: ‘I don’t really go into the [Student’s Union] you know, the bar and that space because it always feels to me like somewhere that’s not for me.’

This need for adequate social space is further explored in *Subtheme B The university’s role*.

*Subtheme 3.3 Problems with accommodation:* Accommodation can both support *(Theme 2)* and hinder *(Theme 3)* social connectedness. Students not living in shared accommodation reported greater difficulty in making connections. Commuter students, for example, miss out on spontaneous social events and can feel disconnected from campus life. Living near campus but alone can also be isolating, even when it is a personal choice:

G2, P3: ‘…I didn’t even have like flatmates or anyone that I could really connect with.’

Conversely, interpersonal dynamics can be challenging when living with strangers in halls of residence, over which students have limited control:

G1, P2: ‘I was put in a particular halls of residence with people are very, very different from me with very, very different interests… because you’re just randomly put together.’

Negative relationships with housemates may be more detrimental to students’ sense of social connectedness than other social ties^57,58^.

*Subtheme 3.4 Interaction anxiety:* Students experience interaction anxiety when meeting new people and/or initiating interactions. Many therefore rely on existing connections,

G3, P1: ‘I would never go to any events that my friends won’t go into.’

even though expanding networks would be preferable *(Subtheme 2.6):*

G2, P3: ‘…we’re kind of kind of sticking to the same groups. And so we’re not really expanding our circles or getting to know people… or expand interests and things like that.’

Alternatively, students turned to social media as a less anxiety-inducing way to establish connections *(Subtheme 2.1)*.

*Subtheme 3.5 Mental health:* Mental (ill)health moderates students’ sense of social connectedness. Even socially connected students can feel isolated if they experience mental ill-health *(Subtheme 1.3):*

G3, P3: ‘…even though I was surrounded by a lot of people, because my mental health was very bad, I felt really not connected to anyone.’

Conversely, positive mental health can increase students’ capacity to engage socially

G3, P3: ‘I struggled with my mental health, but at this point, I was feeling a lot better and able to socialise…’

and social engagement, in turn, can boost mental health:

G2, P4: ‘the ability to actually bump into someone and go for that hot coffee was actually really good element for my mental health.’

This subtheme demonstrates the bidirectional relationship between mental (ill)health and social (dis)connectedness^14,15^.

*Subtheme 3.6 Shortcomings of social media:* Social media is used, and even relied upon, to establish and maintain social connections *(Subtheme 2.1)*, but, paradoxically, it can also hinder social connectedness. During the COVID-19 lockdowns, students missed casual social encounters on campus *(Subtheme 2.3):*

G1, P1: ‘You can’t bump into someone online.’

Outside of lockdowns, maintaining relationships formed online proved difficult for many as they did not always translate into lasting, meaningful connections *(Subtheme 1.3)*:

G3, P2: ‘People that I made connections with, on social media when I was in first year, I very rarely talk to now.’

Students described online interactions as impersonal, superficial, and inferior to in-person interactions. Additionally, students recognised that social media engenders upward social comparison, which amplifies feelings of loneliness and isolation *(Subtheme 1.4)*.

*Subtheme A: The role of the student:* *Subtheme A* spans all three themes. Students recognised that social connectedness requires some level of effort and active engagement. However, some expressed a desire for this effort to be minimal:

G3, P5: ‘I get involved in things when those things are just easy and made simple for me whereas it felt quite effortful for me to do.’

For students who actively engage, it can be frustrating when others do not show the same level of commitment:

G2, P2: ‘I’ve got a [sport] social this evening, and it will be the same five members that turn up every time…’

However, there was also acknowledgement that some individuals may find social engagement more challenging:

G1, P2: ‘I think it must be really tough… if you’re not naturally, an extrovert…’

*Subtheme A* and *Subtheme B* are closely interlinked. While students accept that building connectedness requires effort, the division of responsibility between the university and the individual is difficult to disentangle.

*Subtheme B: The role of the university:* *Subtheme B* also spans all three themes. The university shapes students’ social experience by creating or removing barriers to, and opportunities for, social connectedness. Students hold clear expectations that the university should actively encourage and facilitate social connectedness,

G2, P1: ‘The more we can encourage people, I say we, I mean the uni…’

and to support students who struggle in this regard. Missed opportunities to put on social events were seen as a failing on the part of the university.

Finally, the university can facilitate or restrict social connectedness through its provision of space: students feel restricted by limited opening hours or alcohol-focused venues *(Subtheme 3.2):*

G2, P2: ‘…there’s nowhere to go and sit and have a chat… I find that the campus is actually uninviting in winter [late afternoon/early evening]’

Overall, there is a sense that the university is somewhat responsible for the social connectedness of its students. This responsibility goes beyond providing adequate social events and opportunities and extends to actively encouraging participation, identifying and removing barriers, catering to diverse needs and preferences, and providing ample, accessible, and appealing social spaces.

We now describe the results of Study 2, in which these themes were interrogated in a larger sample via an online survey.

### Study 2 Results - Online survey on experiences of social connectedness and initial app ideas

Table 3 shows the demographic characteristics of the survey sample.

**Table 3:** Survey sample demographics, *n* = 37

Characteristics	*n* (%)
Gender	
Male	7 (18.92%)
Female	29 (78.38%)
Non-binary/third gender	1 (2.70%)
Age	
Mature students	8 (21.62%)
Not mature students	29 (78.38%)
University	
University of Plymouth	36 (97.30%)
Other	1 (2.70%)
Academic year	
Undergraduate first year	4 (10.81%)
Undergraduate second year	10 (27.03%)
Undergraduate third/placement year	3 (8.11%)
Undergraduate fourth/final year	3 (8.11%)
Undergraduate recently completed	4 (10.81%)
Integrated master’s year	1 (2.70%)
Postgraduate taught	9 (24.32%)
Postgraduate research	1 (2.70%)
Postgraduate recently completed	1 (2.70%)
Prefer not to say	1 (2.70%)
Mode of study	
Full time	35 (94.59%)
Part time	2 (5.41%)
Ethnicity	
White	33 (89.19%)
Asian	3 (8.11%)
Other	1 (2.70%)
Reported disability	
Yes	5 (13.51%)
No	32 (86.49%)
LGBTQ+	
Yes	8 (21.62%)
No	27 (72.97%)
Prefer not to say	2 (5.41%)

Participants were asked about their experiences of social connectedness and wellbeing. Participants experienced moderate levels of loneliness (mean UCLA score =47.77, SD = 12.95, range: 24–36, *n* = 42) and wellbeing (mean SWEMWBS = 23.12, SD = 5.04, *n* = 41). There was no correlation between loneliness and wellbeing, *r* = 0.25, *p* = 0.117.

Participants were asked about opportunities and ways to connect. Students (*n* = 42) primarily connected with others via their course (32 instances), accommodation (19), friends of friends (14), societies (13), social media (9), and employment (5).

Participants did not often use campus spaces to socialise (mean = 39.16, SD = 27.42, *n* = 37, scored between 0 – never to 100 – all the time), and were only somewhat likely to attend university social events (mean = 45.33, SD = 23.32, *n* = 36, scored 0 – not at all/never to 100 – definitely/all the time).

Participants were also asked about barriers to participating in social events. Table 4 shows the extent to which barriers identified in Study One made students less likely to attend university social events (from 0 – not at all, to 100 – completely).

**Table 4:** Barriers to attending university social events, *n* = 36

Reason	Mean	St. dev.
Others - not having anyone to go with	63.75	32.97
Location - too far away for me to travel	55.25	37.07
Others - not fitting in with the other attendees	55.17	36.46
Cost - too expensive	54.22	33.80
Location - too difficult for me to get to	48.00	37.38
Cost - a reasonable price but I didn’t/don’t have the money spare	47.28	34.30
Location - not accessible	36.75	39.37
Alcohol - pressure / expectation for me to drink	25.81	35.60
Alcohol - other people would be drinking	21.78	30.24

Most participants felt anxious about social events at university (27/34, 79.41%), causing almost two-thirds (17/27, 62.96%) of whom to ‘miss out’ on social events. As in Study One, students were anxious about meeting new people, especially in large groups,

P25: ‘It was a large group of people and I didn’t know anyone yet. I can feel very anxious around people I don’t know and find it hard to start conversations with people I don’t know.’

in case they did not connect with others or fit in,

P16: ‘Lack of connection, fear of others not liking me’

Several students were anxious due to drinking culture:

P19: ‘Expected to drink with lots of people I didn’t know or knew very little about’

*Social media:* Most students (33/37, 89.19%) used social media to keep up with university news and information and/or to connect with others at university. Yet, views on social media were mixed. Around half of students (24/40, 60%) felt positively towards using social media for university purposes. These students reported that school pages and group chats enabled them to connect and make friends and facilitated communication around group projects and understanding assignments, though there were concerns that social media can replace in-person interactions:

P23: ‘It can be useful for communication during group activities although this can sometimes lead to fewer face-to-face interactions.’

Other participants (14/40, 35%) felt that social media could be helpful if moderated to ensure content remained relevant and users were safeguarded:

P6: ‘When done correctly with the right safeguarding in place it can be very beneficial’

Students noted that alternative options are necessary because ‘…not everyone chooses to have social media…’(P15) and ‘…a lot of people are too nervous to interact’(P13).

Two students (5%) did not agree with using social media for university purposes:

P1: ‘…it makes things more difficult for those who are less confident with technology and opens up less formal communication which some people struggle with.’

Feedback on initial app ideas: Participants rated how successfully the proposed app would improve social connectedness on a scale from 0 (not at all) to 100 (extremely). Overall, students felt the app would be successful (M = 69.41, SD = 15.98. *n* = 32). Most qualitative responses were positive:

P29: ‘I love the idea!’

Students felt the app would facilitate awareness of events,

P17: ‘It’s very easy to miss out on events simply because you don’t even know they are happening’

and increase connectedness,

P6: ‘Can be used to break down barriers, e.g., overcome the difficulties with arranging meeting[s]’

P12: ‘People who might feel alone, won’t have to feel that anymore’

by removing some of the anxiety of initiating connections in-person:

P14: ‘I feel it will help a lot of people’s worries and anxiety around meeting new people / trying things at university’

In this way, respondents thought the app would be at least as effective as social media, but without some of the drawbacks,

P15: ‘More people would willingly download the app as it isn’t a popular toxic social media that many choose not to download’

and with the added benefit of being a one-stop solution:

P28: ‘…it would mean everyone connect[s] in one place rather than across different social media platforms’

However, students remarked that success depends upon ease of use and uptake:

P11: ‘Could be good but I don’t think everyone would bother using it’

P30: ‘Depends if people are willing to download it and the ease of use’

and that anxiety around attending events alone might still be an issue:

P13: ‘It’s a good idea but people may be afraid to attend alone’

One student was concerned about stigma,

P38: ‘…people might be worried that [they would] be negatively labelled for having to use an app to make friends’

and another about inclusivity and representation:

P42: ‘My age and type of degree probably wouldn’t be represented.’

Overall, participants were fairly likely to use the interactive map feature (M = 62.06, SD = 24.71, *n* = 32, where 0 = ‘not at all’ and 100 = ‘extremely). Participants also ranked the usefulness of 11 other features. Most often ranked first place was a forum/message board, followed by mental health signposting (2^nd^ place, 3^rd^ place), and psychoeducation (4^th^ place). Student suggestions included event reminders, attendee limits, online events, societies pages, and a ‘similar symptoms/situations board to show others feel the same’.

We now describe Study Three, in which findings from Studies One and Two were fed forward into iterative co-production workshops with students.

### Study 3 results - iterative think-aloud co-production workshops

In the first co-production workshops, participants co-designed a functional specification for MAPP based on findings from previous studies. Workshop notes were organised into four themes (see Methods section for further details).

**Theme 1 Potential benefits:** Overall, students were enthusiastic about the app and its central map feature. A key benefit identified was the provision of clear and immediately accessible information about happenings without needing to be on campus. The map was also expected to facilitate navigation, which would benefit (particularly new) students who find navigating the campus *‘daunting and confusing’*. Knowing about events in advance was also expected to enable planning, encourage trying new things, enhance motivation to spend more time on campus, and make commuting to campus *‘feel more worthwhile’*. Students also liked having university social information on one platform, removing the need for social media.

**Theme 2 Features**: Five additional features and functionalities were suggested.

*Subtheme 2.1 Messaging and social media:* Students suggested that event-related message boards would facilitate coordination and that it would be helpful *‘to see who else may be going’*. Some students also wanted private and group chat functions, though other students expected to use *’other social media for messaging‘* friends generally, and in-app messaging only if they were attending an event alone. One group felt that a ‘like’ feature would be *‘toxic… [and] a breeding ground for comparison, which can make app users feel worse’*. Some students suggested the option to link social media profiles to their MAPP accounts.

*Subtheme 2.2 Filters:* Students suggested the ability to personalise the map through coloured filters to prevent overwhelm. Suggestions for filters included topic, course, society, placements, and whether events were alcohol-free. Students also wanted to control who could see their posts by filtering by demographics or group membership.

*Subtheme 2.4 Group working:* Some students suggested that MAPP should facilitate collaboration with coursemates by enabling them to share Google Docs and OneDrive files.

*Subtheme 2.5 Calendar and reminders:* Calendar and reminder functions were suggested to help students manage their time.

*Subtheme 2.6 Wider community:* Students suggested that MAPP’s scope could be extended beyond the campus into the wider community because *‘a lot of social events don’t necessarily happen on campus itself’*.

**Theme 3 Safety concerns:** The main concerns students raised were around safeguarding. It was important for students to control who could see their activity. For students advertising events, unwelcome attendees were a concern. Examples included *‘if [a student] was LGBT and didn’t feel comfortable advertising a small-scale event to everyone due to the risk of homophobic people turning up’* and *‘[wanting a] fellow female to walk home with them late at night.’* The proposed solutions were a filter function *(Subtheme 2.2)* and to verify users’ identities with university credentials, which would also facilitate moderation.

**Theme 4 App design and usability:** For wide adoption, it was considered essential that the app is easy to use, navigate, and read, does not store unnecessary data nor crash frequently, is kept up to date, and has a modern, simple, and gender-neutral aesthetic using a ‘flat’ illustration style.

In the final workshop, participants gave feedback on the mock-ups produced by the URAs. Workshop notes were organised into three themes (see Methods section for further details).

**Theme 1 Map feature:** Students liked and would use the interactive map, but they felt clearer guidance was needed around creating groups, posts or events, user permissions, and moderation.

*Subtheme 1.1 Safety and privacy:* Students wanted an anonymous *‘ghost mode’* to enable them to use the app without their activity being publicly visible.

*Subtheme 1.2 Map design and features:* Students felt a 3D map that *‘looks like a map’* with a compass, labelled buildings, and current location marker would make MAPP user friendly and easy to navigate. Additional suggestions included adding timings to event pins, and identifying *‘welfare points, sustainability areas, cash points, period boxes [free menstrual products]’* on the map.

**Theme 2 Additional features:** Students proposed filters for forums and live chats (by cohort, subject, etc.); user profiles; a safety page; a calendar; and a link to the university app. Students also suggested including other local post-secondary institutions to enable broader connections.

**Theme 3 General design and usability:** Design suggestions included a bright interface, larger and clearer text, and clearer labelling of pages and functions. For example, students were unsure whether the ‘general info and resources’ section referred to MAPP or to the university more generally.

## Discussion

This project explored students’ experiences of loneliness and social connectedness at university, and co-designed a digital health solution. Using the principles of participatory action research, each study was designed, conducted, and analysed by undergraduate students supervised by a postgraduate and one academic. Study One employed focus groups to investigate experiences of loneliness, belonging, and social connectedness at university. Study Two further interrogated resultant themes in an online survey. Study Three used these findings to co-design a solution. This discussion summarises and then synthesises our findings, focusing on loneliness as a systemic issue and evaluating the potential of the co-designed solution.

**In Study One,** students participated in focus groups about their experiences of loneliness and social connectedness. Our participants felt that being socially connected was necessary but not sufficient to preclude loneliness, supporting previous findings^59^. There also needs to be a sense of shared experience and belonging, which can be cultivated through course affiliation, group academic activities, and using the campus, where academic, social, and potentially living spaces are shared. These routes to social connection are passive, as they involve connecting with exogenous (pre-determined) groups of coursemates or housemates, piggybacking social plans onto timetabled activities, or simply absorbing the bustle of the campus. More active ways to connect include joining societies or other endogenous (self-selected) groups, attending social events, and widening networks via employment or community engagement.

The distinction between active and passive social connectedness is interesting. This thread weaves through all three themes and becomes explicit in the two overarching subthemes concerning the roles of students and the university. While students accepted some responsibility for their social experience, they also expected the university to facilitate social connectedness and to support students who struggle to engage. Specifically, universities should mitigate barriers affecting students who commute to campus or experience disability, financial hardship, mental ill-health and/or social interaction anxiety. More generally, students expect the university to promote and clearly communicate accessible and inclusive social opportunities, to minimise the effort required of students, and to provide adequate social space.

Campus space is one of the main threads interlinking the themes. It is here that experiences are shared, that students find ‘some*where* [they] fit in’, and that connections form, passively or actively. Accordingly, a familiar, accessible, inclusive and inviting, versatile and functional campus, bustling with varied social opportunities, is itself conducive to the sense of belonging necessary to promote social connectedness and preclude loneliness. Conversely, connectedness may be hampered by a campus that feels unfamiliar, uninviting, inaccessible, or exclusive, or whose social spaces are time-restricted, scarce, unfit for purpose, or tailored towards particular groups or preferences. There is, therefore, a need for adequate ‘third places’ on campus – spaces dedicated to socialising rather than living or studying^60^. However, bars and pubs are not sufficient, as drinking culture was a barrier for many. The conclusion that shared space is central to students’ social connectedness is reinforced by the finding that closing university campuses during the COVID-19 pandemic severely impeded students from forming, building, and maintaining social connections^61^.

The disruptions to social connectedness caused by campus closures occurred despite online connectivity, suggesting that online interactions are no replacement for in-person interactions. Social media is central to students’ social lives^27,55^, and allows students to avoid the anxiety experienced when initiating connections in-person. However, some students struggle to initiate and maintain connections online, and these relationships may be perceived as lower quality or less fulfilling. We surmise that online connectedness via social media not only fails to provide the sense of shared experience and belonging necessary to preclude loneliness, but increases it by stimulating fear of missing out^62,63^ and upward social comparison^64,65^. Feelings of loneliness and disconnectedness can be amplified by logging on to see seemingly highly socially connected peers appearing to have the time of their lives.

To conclude, shared experience and belonging are paramount in students’ social connectedness, for which the institution, as well as the individual student, is responsible. The perceived onus on ‘the university’ supports our argument that student loneliness is not an individual issue but a systemic one, and the focus on shared experience and belonging, underpinned by group membership and shared campus space, aligns with the theories discussed earlier. Students must feel they belong to university communities (self-determination theory), with available and accessible shared networks and resources, including the campus itself (social capital theory). Moreover, as per symbolic interactionism theory, the campus acts as a shared symbol, representing both community belonging and access to shared resources. Therefore, a digital health solution could tackle student loneliness systemically rather than individualistically by basing itself not around individual profiles, connections, or interventions, but on the existing, place-based community symbolised by the campus itself. As such, we conceptualised MAPP: a student social app based around a dynamic and interactive map of the university campus displaying live events and community happenings.

**In Study Two,** a larger sample of students participated in an online survey about experiences of loneliness and social connectedness, and initial app ideas. Study Two supported Study One’s findings regarding opportunities for, and barriers to, connection. Participants most commonly made friends via their course, a key opportunity for connection in Study One. Study One showed that students who use social spaces and attend university social events regard the campus as a source of connectedness, but Study Two showed that students are relatively unlikely to do so. This indicates not only a need for adequate ‘third places’ on campus^60^, but sufficient scaffolding to enable and encourage students to make use of them. Therefore, we hypothesise that interventions to encourage more students onto campus will create a positive cycle; the more often a student comes to campus the more connected they will feel, and their presence on campus will contribute to the busy, vibrant atmosphere that will in turn attract more students onto campus. Other key opportunities for connection identified in Study One and supported by Study Two include accommodation, societies, social media, and employment.

Of the barriers to connection identified in Study One, the most highly rated reasons to avoid social events were having to go alone, travel, not fitting in, and cost. Belonging and fitting in were strong threads in Study One, as were financial concerns, difficulties experienced by commuter students, and interaction anxiety. Also aligning with Study One, the majority of Study Two participants felt anxious about social events, often due to concerns around meeting new people, fitting in, and drinking culture. These concerns are well-represented in the literature, with students reporting financial stress^66^ and high levels of anxiety^8^. However, the finding that excessive drinking as a social practice^56^ may be in decline among university students may represent a novel finding, aligning with broader evidence of declining alcohol consumption among young people^67^.

To mitigate social anxiety, Study One participants used social media, particularly to form new connections. However, they concurrently experienced social media as a barrier to social connectedness. Study Two aligned with Study One in this regard and reflected students’ well-established reliance on social media^27^. However, opinions were again mixed; around half endorsed using social media for university purposes while others had concerns around relevancy of content, safeguarding, and the risk of it replacing in-person interaction.

The motivation behind MAPP is to retain the benefits of social media while addressing these shortcomings. Qualitative responses suggested that a university-managed platform could improve social connectedness without the drawbacks of conventional, ‘toxic’ social media. Accordingly, most students agreed that the app would be successful in this regard, and would likely engage with the central, interactive map feature. However, concerns about stigma indicate misunderstanding of the app’s purpose; rather than being a mental health intervention or tool solely for individuals in need of friendships, MAPP is designed to be a universal platform for accessing social events and connecting with the university community. Nevertheless, appeal, uptake, and inclusivity are key concerns deserving special attention.

Studies One and Two identified gaps in social connectedness at university. **Study Three** employed co-production workshops to shape a student-led solution. Based on findings from Study One, Study Two, and the initial Study Three workshops, the URAs produced an interface design mockup for evaluation in later Study Three workshops. This iterative approach facilitated refinement based on direct stakeholder feedback, resulting in a final functional specification that accurately reflects and fulfils user needs and preferences.

Participants expressed enthusiasm for MAPP’s potential benefits, particularly the central map feature, which was expected to simplify navigation and improve awareness of campus events. This feature was anticipated to be especially beneficial for new students in reducing overwhelm. Other anticipated benefits included having all university social information on one platform and reducing reliance on social media.

In addition to the map, students suggested features including message boards, filters, information provision, group working tools, and calendar functions. Safety concerns were prominent; students emphasised the need for user verification, moderation, and control over privacy and visibility settings. These features should be intuitively contained within an app that is easy to navigate, visually appealing, and inclusive.

Centring the app around a campus map disrupts the conventional structure of social media, shifting the focus from individual profiles to the system as a whole. This shift aligns with the argument that loneliness is a systemic problem rather than an individual one. Our findings support this argument in several ways. First, students feel that the university plays an integral role in their social connectedness. The institution facilitates passive social connectedness by enrolling students into exogenous groups (e.g., course or accommodation), and by providing shared space (the campus). While students accept some responsibility to engage socially, they also expect the university to actively encourage connection and to support students who struggle to connect. Second, the importance of shared campus space was a key finding. A familiar, accessible, and inclusive campus fosters shared experience, connectedness, and belonging, whereas an unfamiliar, inaccessible, or exclusive campus hinders connectedness. Third, the systemic disruptions caused by COVID-19 significantly impeded social connectedness by rendering the campus inaccessible. Finally, systemic problems require systemic solutions, and students already use social media to engage with university networks.

Concerns around social media included lack of appropriate moderation, that it can replace in-person interaction, and that students who choose not to use ‘toxic’ social media (which can cause upward social comparison and FOMO^42,62,63,65^ would miss out on important university information and opportunities. Students also raised concerns that align with digital anxiety, such as discomfort with online social interaction and fears around privacy and visibility. MAPP’s design directly responds to these concerns. The key output from this project is a blueprint for a digital solution that maintains the benefits of social media and avoids these drawbacks by adopting a wholly systemic approach. These solutions are discussed in the following section.

MAPP would enable students to connect with university communities on a centralised, purpose-built system that incorporates safeguarding into its design, that facilitates, rather than replaces, face-to-face interaction, that removes the need for additional platforms, and that does not encourage upward social comparison^64,65^ or facilitate problematic endless scrolling^68^. Rather, it would leverage the significance of shared campus space, in a ‘psycho-geography style way’ (Study One), by making the campus map a visual symbol of community, social capital, living social networks, and shared resources^1,37,38^ that is appealing, inclusive, and accessible to students even when they are not physically present. As such, MAPP not only facilitates both passive and active social connection but uses the former to scaffold the latter. Connections to exogenous groups, including courses, societies, and the university itself, are made explicit and instantly accessible. We hypothesise that this increased community visibility and accessibility will instil a sense of belonging that will support students in actively engaging socially (i.e., initiating endogenous connections).

MAPP’s shift in focus from the individual to the system has a second key advantage. It provides an overview of university social networks, activities, and events that can be used by institutional leaders to ensure inclusivity, monitor the use of campus space, and identify gaps in provision.

The stakeholder support presented here indicates that app development is a worthwhile next step. However, several important considerations should be addressed. For example, understandings of loneliness, belonging, and social connectedness vary within the literature^69^, and Barreto et al. (2024, p.167)^14^ warn against ‘romanticizing communities’ without critical appraisal of what these communities are, what they stand for, and the norms and narratives they perpetuate. The complex nature of these concepts highlights the need for more precision in how they are defined and operationalised in the design and evaluation of MAPP.

Another construct of interest, fear of missing out (FOMO) is a well-documented problem associated with social media^62,63^. MAPP’s success is contingent upon creating a supportive and inclusive environment for social connectedness without fostering anxiety around missed opportunities. This issue will require careful consideration, with stakeholder input, throughout the design and development process.

In addressing these challenges, further studies would benefit from larger sample sizes, especially when evaluating the efficacy of the app. However, it is essential to remember the diversity of MAPP’s user-base and to continue to prioritise issues of equality, diversity and inclusion. Though this diversity was reasonably well represented here, future stages of co-creation should actively seek input from a representative stakeholder group, focusing on underrepresented ethnic groups and students from a broader range of universities. MAPP aims to make the university community visible and accessible, and it is essential that marginalised groups feel equally included. Therefore, universities must actively identify and mitigate barriers to inclusivity—both within the app and the university more generally. Relatedly, university leaders and professional services staff are key stakeholders who should be included in the co-creation process.

Although this project has not yet progressed to app development, the extensive elicitation and design work offered important insights into how PAR can support systemic, rather than individualistic, approaches to student loneliness. Working with URAs enabled the team to surface perspectives that would likely have been missed in expert-led design; their experiences enriched the reflexive qualitative analyses; and they helped create psychologically safe research environments where participants could share openly. At the same time, challenges of PAR included the time, labour, resources, and coordination required, specifically in funding a team of URAs and ensuring they had adequate supervision. If MAPP is taken forward, the PAR process will need to evolve to include a wider stakeholder base (e.g., underrepresented student groups) and to embed ongoing participatory cycles throughout app development and implementation. Crucially, the approach must continue as a way to share power authentically in mental health-related problem-solving endeavours. In this sense, the method is not just a means to co-create an intervention but is itself part of the systemic shift we argue for: a move from top-down, expert-defined, individualistic solutions to critical evaluation of the system led by those who live and work within it.

In conclusion, our findings demonstrate that student loneliness can be experienced and understood systemically, shaped not only by individual behaviour but also by the social and physical structures of the university. Students conceptualised connectedness as something that institutions actively enable through space, opportunity, and inclusive design. Through our PAR approach, these systemic dynamics directly informed the co-creation of MAPP. MAPP is not an intervention for lonely individuals. Rather, it is a digital solution that intentionally shifts away from individualistic interventions towards a platform that makes the system itself visible, accessible, and navigable. It achieves this by visualising and representing the university campus as a shared physical resource and symbol of community and social capital. As MAPP progresses towards development, future cycles of co-creation will need to expand to include more diverse student voices and institutional stakeholders. MAPP should retain its systemic focus by supporting universities to understand, monitor, and cultivate the conditions that foster student social connectedness.

## Supplementary information

Supplementary information

## Data Availability

Data have been deposited on ReShare: 10.5255/UKDA-SN-856114.
