# Supplementary material for: Designing a systemic intervention for student loneliness and social connectedness using a mixed-methods, co-creation approach
Source: Npj Ment Health Res. 2026 Feb 21;5:12. doi: 10.1038/s44184-026-00191-9 (PMC12924778; doi:10.1038/s44184-026-00191-9)
Supplement: Supplementary file 1 — Supplementary information [file 44184_2026_191_MOESM1_ESM.pdf]

Focus Group Topic Guide

1. **We are interested in students' experiences of social connectedness at university. What does it mean to be socially connected as a student?**
   Prompts: How would you define social connectedness? How would you explain it to someone else? How would you explain it to a non-student?
   Has this changed since the pandemic?

2. **Thinking back to before the pandemic, we're interested in your experiences of socialising at university. What made you more likely to go to an event on campus, or use a communal space, or join a society? What made you less likely to do those things?**
   Prompt: If you were aware of an event on campus, like a campus market or a coffee morning, what would be the main thing stopping you from going?

3. **What are your experiences of making friends at university?**
   Prompts: How easy / difficult was it to make friends? When did you make your friends? (e.g. start of term, later on the term)

4. **Could everybody describe a time they felt socially connected at university?**
   Prompts: What was that like? What was it that made you feel socially connected? How did it feel?
   Before and after the pandemic?

5. **Could everybody describe a time they did not feel socially connected at university?**
   Prompts: What was that like? What was it that made you feel disconnected? How did it feel?
   Before and after the pandemic?

6. **What sort of things promote social connectedness at university?**
   Prompts: Accommodation, academic activities, university-based social activities, sports, Student Union, clubs and societies, culture
   Has this changed since the pandemic?

7. **What sort of things cause disconnectedness at university?**
   Prompts: Accommodation, academic activities, university-based social activities, sports, Student Union, clubs and societies, culture
   Has this changed since the pandemic?

8. **Thinking back to when you first started university and/or returned for Stage 2, Stage 3, etc. what were your social experiences around the start of term?**

9. **What could the university do to improve social connectedness in students?**
   Prompts: Accommodation, academic activities, university-based social activities, sports, Student Union, clubs and societies, culture
   Response to the pandemic
